# A Prospective, Multicenter Randomized Controlled Trial (RCT) of the da Vinci SP^®^ Surgical System versus Open Surgery in Nipple-Sparing Mastectomy (NSM) Procedures for Patients with Breast Cancer

**DOI:** 10.1245/s10434-026-20201-6

**Published:** 2026-07-16

**Authors:** Katherine Kopkash, Mara Piltin, James Jakub, Sara Javid, Meghan R. Flanagan, Rebecca Aft, Akiko Chiba, Alysandra Lal, Ari D. Brooks, Farin Amersi, Nayana Dekhne, Jennifer Tseng, Clara Farley, Alan Kadison, Jeffrey Johnson, Rosa F. Hwang

**Affiliations:** 1 Department of Surgery, Endeavor Health, Evanston, IL USA; 2 University of Chicago Pritzker School of Medicine, Chicago, IL USA; 3 Department of Surgery, Mayo Clinic, Jacksonville, FL USA; 4 Department of Surgery, University of Washington, Seattle, WA USA; 5 Department of Surgery, Washington University, St. Louis, MO USA; 6 Department of Surgery, Duke University, Durham, NC USA; 7 Department of Surgery, Ascension Columbia St. Mary’s Hospital, Milwaukee, WI USA; 8 Department of Surgery, University of Pennsylvania, Philadelphia, PA USA; 9 Department of Surgery, Cedars-Sinai Medical Center, Los Angeles, CA USA; 10 Department of Surgery, Corewell Health, Royal Oak, MI USA; 11 Department of Surgery, City of Hope Comprehensive Cancer Center, Irvine, CA USA; 12 Department of Surgery, Emory University, Atlanta, GA USA; 13 Department of Surgery, Northwell Health, Lake Success, NA USA; 14 Department of Surgery, Mayo Clinic, Rochester, MN USA; 15 Department of Breast Surgical Oncology, University of Texas MD Anderson Cancer Center, Houston, TX USA; 16 New York University Grossman Long Island School of Medicine, Mineola, NY USA

## Abstract

**Background:**

This prospective randomized trial evaluated the safety and effectiveness of robotic-assisted nipple-sparing mastectomy (rNSM) using the da Vinci Single Port (SP)^®^ compared with open NSM in women with early stage breast cancer.

**Patients and Methods:**

This Food and Drug Administration (FDA) investigational device exemption (IDE)-approved, two-arm, multicenter randomized controlled trial (RCT) randomized eligible women (clinical Tis-T2, N0) 1:1 to rNSM or open NSM with immediate implant-based reconstruction. Operations were performed at 14 US sites by 23 surgeons. Primary endpoints included conversion to open surgery, adverse events at 42 days, and positive surgical margin (PSM) rates. Secondary outcomes included operative time, estimated blood loss, nipple–areolar complex (NAC) and skin flap viability, final pathology, and patient-reported outcomes (BREAST-Q).

**Results:**

Overall, 74 patients completed surgery, totaling 65 rNSM (38 therapeutic, 27 prophylactic) and 66 open NSM (38 therapeutic, 28 prophylactic). Operative time was longer for rNSM (141.6 versus 83.9 min) with no conversions to open surgery. Serious adverse events (SAEs) occurred in three rNSM and eight open NSM patients, with no device-related SAEs reported. NAC preservation at 42 days was 100% for rNSM and 98.5% for open NSM. PSM rates were identical (six patients each). Higher BREAST-Q scores favored rNSM in multiple domains.

**Conclusions:**

These results demonstrate that da Vinci SP^®^ rNSM is feasible and has a safety profile comparable to open NSM for early stage breast cancer, with similar margin status and postoperative complications. Although SAEs and patient-reported outcomes were more favorable with rNSM, these differences were not statistically significant. rNSM was associated with longer operative times, which may in part reflect early experience with the technique.

**Supplementary Information:**

The online version contains supplementary material available at https://doi.org/10.1245/s10434-026-20201-6.

Nipple-sparing mastectomy (NSM) has become an important surgical option for selected patients with breast cancer, offering excellent oncologic outcomes while preserving the native skin envelope and nipple–areolar complex (NAC) to optimize cosmetic results. Although NSM is widely accepted, it remains technically demanding and associated with increased surgeon workload, poor ergonomics, and surgeon fatigue compared with a skin-sparing or total mastectomy. NSM also has a risk of NAC ischemia or necrosis and mastectomy skin-flap compromise.^1–3^^,^^4^ As a result, there has been growing interest in alternative approaches that may improve flap perfusion, reduce morbidity, improve surgeon ergonomics, and enhance cosmetic and patient-reported outcomes.

Robotic-assisted nipple-sparing mastectomy (rNSM) was introduced as a minimally invasive alternative to open NSM, with early studies demonstrating feasibility and short-term safety.^5–7^ International experience with rNSM in Europe and Asia has accelerated, and emerging data suggest rNSM may offer advantages in complication rates, cosmetic outcomes, sensation preservation, and surgeon ergonomics.^5–10^ However, follow-up is limited, and long-term oncologic outcomes are still maturing. To date, only a single randomized controlled trial has compared rNSM with open NSM, reporting similar complication rates and improved patient-reported outcomes with rNSM but longer operative times and relatively short oncologic follow-up.^7^

Despite the growing experience globally, robust clinical trial data for rNSM are limited, especially within the USA. We conducted a prospective, two-arm multicenter randomized controlled trial (RCT) comparing robotic and open NSM in women with early stage breast cancer in the USA. The objective of this RCT was to compare the safety, perioperative outcomes, and oncologic effectiveness of rNSM with open NSM.

## Patients and Methods

### Study Design

**Fig. 1:**
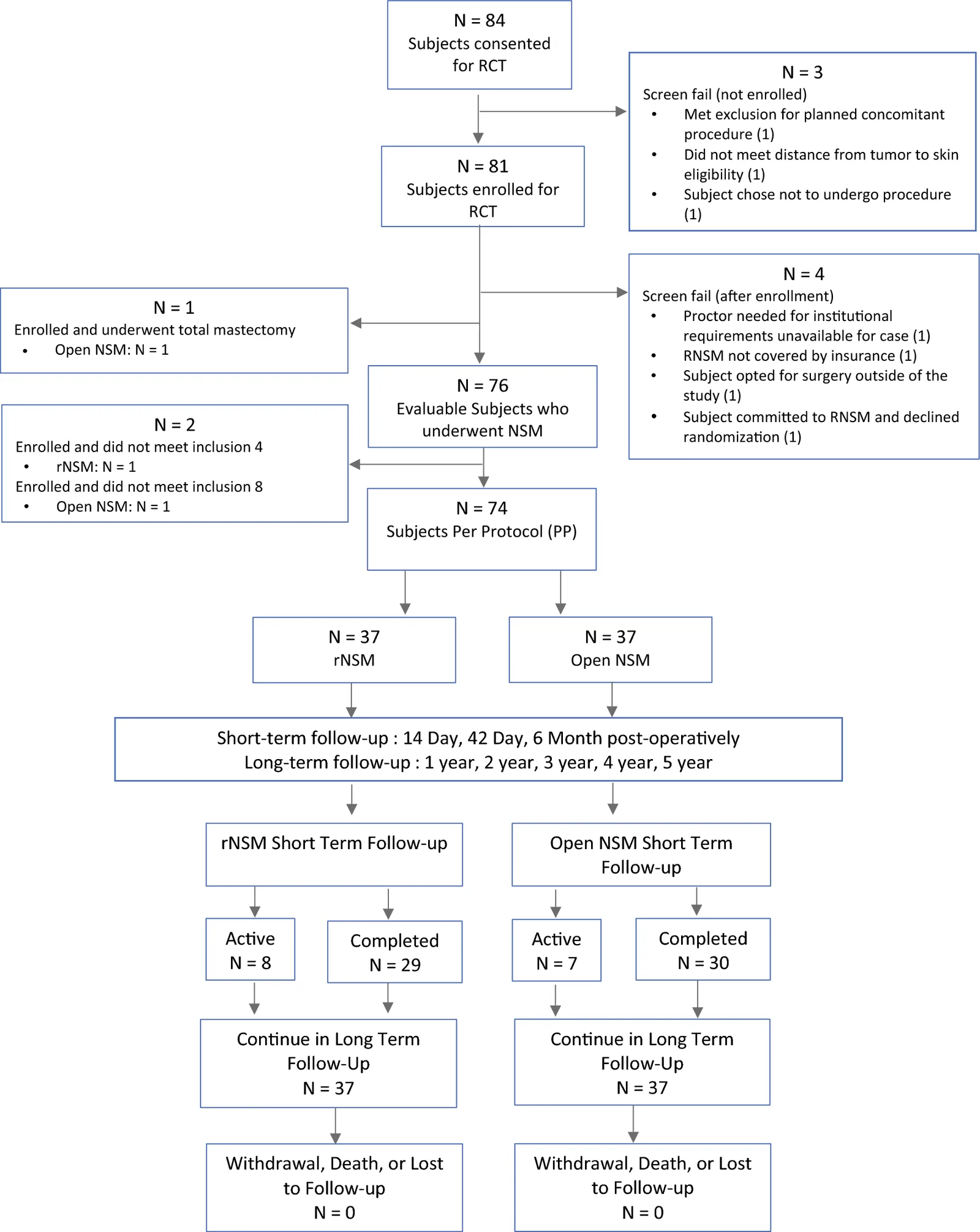
CONSORT flow diagram of patients undergoing rNSM versus open NSM

This study was a prospective, two-arm, multicenter randomized controlled trial (RCT; Food and Drug Administration [FDA] investigational device exemption [IDE] G220319; NCT03892980). Operations were performed at 14 US sites after institutional review board (IRB) approval by 23 surgeons who had undergone specific training in the use of the SP system, the study protocol, and the study procedures. The study was structured in two phases: an initial roll-in phase followed by the RCT phase. The roll-in phase allowed each participating site to enroll up to two nonrandomized subjects to undergo rNSM to gain experience with the investigational device and the procedure. The roll-in subjects were excluded from the safety analysis of the randomized cohort and were analyzed independently. An independent breast surgeon not affiliated with the participating institutions or the sponsor who was familiar with rNSM served as the data safety monitor (DSM). The study sponsor (Intuitive Surgical Inc.) provided the investigational device and technical training but had no role in data analysis, interpretation, or manuscript preparation. This randomized trial was conducted and reported in accordance with the Consolidated Standards of Reporting Trials (CONSORT) guidelines (Fig. 1 and Supplementary Table 1).

### Eligibility Criteria

Study patients were women with early stage breast cancer (clinical Tis-T2 and N0), body mass index (BMI) < 30 kg/m^2^, tumor–skin distance ≥ 5 mm, tumors located ≥ 10 mm from the nipple–areolar complex (NAC), calcifications ≤ 5 cm for ductal carcinoma in situ (DCIS), breast size C cup or smaller, breast ptosis ≤ grade 2, and candidates for unilateral or bilateral NSM procedure(s) (Supplementary Fig. 1). Clinical evaluations, including breast imaging, were performed at the surgeons’ discretion. Contralateral prophylactic NSM and neoadjuvant treatment were allowed. Immediate reconstruction was performed as one- or two-stage implant-based procedures.

After informed consent was obtained, patients were randomized 1:1 to rNSM or open NSM. Randomization was performed by a randomization schedule assigned at each site through the central electronic data capture system. Enrollment personnel did not have access to the random allocation sequence. Randomization was assigned at the preoperative visit, approximately 2 weeks before surgery, and without blinding of subjects, surgical teams, or research coordinators. Block randomization was stratified by surgeon and by unilateral or bilateral procedure. The size of the randomization block was 2, 4, or 6 and was determined to ensure a balanced 1:1 ratio between robotic and control arm at a site and procedure level. A database file with the sequential number randomization assignment was provided by the study sponsor to each site upon receiving IRB approval of the study.

### Surgical Technique

Open NSM was performed per the operating surgeon’s standard technique, with most patients having an inframammary fold incision; however, one patient had an elliptical incision, and four patients had radial incisions. For rNSM, the patient was positioned with their lateral chest at the edge of the table for the operative breast, the boundaries of the breast were marked on the skin, and an incision was made at the level of the NAC in the anterior axillary line, spanning 3–4 cm. To adequately allow full insertion of the SP access port wound retractor, a 5-cm semicircle was dissected in an open fashion. The breast tissue was first dissected from the skin and subcutaneous fat anteriorly and then separated from the pectoralis muscle, including the pectoralis fascia posteriorly. The anterior and posterior dissection planes were connected superiorly and inferiorly, releasing the lateral breast gland.

The da Vinci SP Access Port Kit wound retractor was inserted through the incision, and pneumocavity was established to a pressure of 8–12 mmHg. The patient’s ipsilateral arm was secured overhead in a neutral position, and the operating room table was tilted 10–20° to elevate the ipsilateral breast. The da Vinci SP cannula mount was docked to the da Vinci SP Access Port, the endoscope was connected, and the da Vinci SP Monopolar Curved Scissors and Fenestrated Bipolar Forceps were connected for the dominant and nondominant hands of the surgeon, respectively. The surgeon could use a third da Vinci SP instrument for additional retraction, if desired.

The remainder of the breast gland was dissected from the posterior plane followed by the anterior plane. Flap thickness was assessed intraoperatively for rNSM using magnified visualization of the breast tissue with the SP camera and transillumination of the flap. After undocking, the specimen was removed en bloc through the wound retractor with extension of the incision as needed. Sentinel lymph node biopsy was performed per surgeon’s standard open technique through the same incision. Reconstruction was performed non-robotically through the same incision. Drains were routinely placed.

### Study Endpoints

The primary outcomes of interest were conversion from rNSM to open NSM, adverse event (AE) rates at 42 days postoperatively, and positive surgical margin (PSM) rates. The PSM was defined as ≤ 2 mm for DCIS and “ink on tumor” for invasive cancer on the final pathology report.

Data on AEs were collected intraoperatively, at postoperative day (POD) 14, POD 42, 6 months, and annually for years 1–5. Serious adverse events (SAEs) were defined as: any AE that (a) led to death, (b) led to serious deterioration in health, that either: (1) resulted in life-threatening injury, or (2) resulted in permanent impairment, or (3) required inpatient hospitalization, or (4) resulted in an intervention to prevent life-threatening injury or permanent impairment, or (c) led to fatal injury that resulted in death.

The NSM procedure time was analyzed per breast and defined as the time from incision on the breast until the breast specimen was removed from the body. The reconstruction procedure time was defined as the time from when the plastic surgeon began reconstruction until the skin closure completed. Skin flap viability was documented at the 42-day follow-up visit by the surgeon via the Skin Ischemia and Necrosis (SKIN) score. The SKIN score is a validated tool^11^ to assess the severity of mastectomy skin flap necrosis (MSFN) by evaluating both the depth (A–D) and surface area (1–4) of tissue injury.

Patient-reported outcomes were collected using the BREAST-Q questionnaire,^12^ which was administered preoperatively and postoperatively at 42-days, 6 months, and 1 year. The scales utilized included Psychosocial Well-being, Sexual Well-Being, Satisfaction with Breasts, Physical Well-Being (chest), Satisfaction with Back, and Physical Well-Being (arm swelling). NAC sensitivity using a 5-point scale was assessed at 42 days postoperatively as well as 6 months and 1 year.^13^

### Statistical Considerations

This study was designed as a noninferiority trial. The sample size for this randomized controlled study was based on the co-primary safety endpoint of breast procedure-related composite adverse event rates reported within 42 days following the procedure. The composite complication rate for open NSM procedures is estimated around 26% in the literature, so a minimal sample size of 78 subjects per arm (156 subjects for both arms) provides at least 90% power at the 20% one-side alpha level for the co-primary safety endpoint to evaluate the noninferiority of the SP NSM procedure over the open NSM procedure with a margin of 15% using a *Z*-test of unpooled variance*.* Baseline characteristics of rNSM versus open NSM groups were compared using Welch’s two-sample *t*-test, Pearson’s chi-squared test, or Fisher’s exact test, depending on the variable type and distribution. Two-sided *α* level of 0.05 was used to determine statistical significance. SAS version 9.4 software was used for statistical analysis. Enrollment of new subjects was terminated early after FDA 510(k) clearance was granted. Follow-up of all enrolled participants continues per protocol. A post hoc exploratory analysis was performed of the learning curve by comparing operative times of surgeons’ first and third rNSM.

## Results

A total of 74 patients were consented, enrolled, and randomized, and had their surgery completed per protocol between 11 August 2023 and 17 December 2025 (Fig. 1). The groups were evenly distributed, with 37 patients undergoing 65 rNSM (38 therapeutic mastectomies, including 1 patient with bilateral breast cancer; 27 contralateral prophylactic mastectomies [CPM]) and 37 patients undergoing 66 open NSM (38 therapeutic mastectomies, including 1 patient with bilateral breast cancer; 28 CPM).

Patient and tumor characteristics were similar between groups (Table 1). Mean age was 49.6 years for rNSM versus 46.2 for open NSM (*p* = 0.2), and mean BMI was 22.0 versus 23.1, respectively (*p* = 0.1). Most patients had B-cup breast size (64.9% rNSM versus 51.4% open NSM). Mean tumor size was similar (1.8 cm versus 1.9 cm; *p* = 0.9), and all patients were clinically node negative. A sentinel lymph node biopsy was performed on the side of the cancer for all patients at the time of mastectomy. The most common histology in both groups was invasive ductal carcinoma (60.5% rNSM and 65.8% open NSM). There was no significant difference in tumor-to-skin distance on breast imaging (13.7 mm for rNSM versus 15.6 mm for open NSM; *p* = 0.4) or tumor-to-NAC distance (36.3 mm for rNSM versus 34.8 mm for open NSM; *p* = −0.7). Neoadjuvant therapy was administered in five patients (13.5%) undergoing rNSM and ten patients (27.0%) undergoing open NSM (*p* = 0.15). There was no difference in median follow-up (201 days rNSM versus 188 days open NSM; *p* = 0.30).

**Table 1:** Patient demographics and tumor characteristics

**Characteristic**	**rNSM**	**Open NSM**	***p*-Value**
Number of subjects	37	37	
Number of breasts	65	66	
Age, years; median (range)	48 (29–69)	46 (21–71)	0.2
BMI, kg/m^2^; mean (range)	22.0 (17.9–29.0)	23.1 (17.1–28.9)	0.10
Breast cup size			0.4
A	7 (18.9)	7 (18.9)	
B	24 (64.9)	19 (51.4)	
C	6 (16.2)	11 (29.7)	
Ptosis			0.7
Grade 0	10/37 (27.0)	9/37 (24.3)	
Grade 1	21/37 (56.8)	19/37 (51.4)	
Grade 2	6/37 (16.2)	9/37 (24.3)	
Indication for NSM			> 0.9
Therapeutic	38 (58.5)	38 (57.6)	
Prophylactic	27 (41.5)	28 (42.4)	
Clinical stage*			0.7
Tis	8 (21.1)	9 (23.7)	
T0	0 (0.0)	1 (2.6)	
T1	19 (50.0)	15 (39.5)	
T2	11 (28.9)	13 (34.2)	
N0	38 (100)	38 (100)	
Tumor histology*			
DCIS only	8 (21.1)	10 (26.3)	0.788
IDC	23 (60.5)	25 (65.8)	0.812
ILC	6 (15.8)	3 (7.9)	0.480
Other	1 (2.6)	0 (0.0)	> 0.9
Tumor size*, cm; mean (SD)	1.8 (1.09)	1.9 (1.36)	0.9
Tumor-to-skin distance*, mm; mean (SD)	13.7 (8.27)	15.6 (10.87)	0.4
Tumor-to-NAC distance*, mm; mean (SD)	36.3 (19.05)	34.8 (17.52)	0.7
Neoadjuvant therapy	5 (13.5)	10 (27.0)	0.15
Follow-up (days)^†^			
Median (IQR)	201 (165–377)	188 (168–296)	0.30
Mean (SD)	256.8 (162.2)	209.1 (118.8)	
Min–max	35–652	37–466	

^*^Only applicable for therapeutic breasts; analysis performed at breast level

^†^Follow-up time is defined as the number of days from subject’s date of surgery to their last follow-up visit

Numbers are No. (%) unless indicated otherwise

*NSM* nipple-sparing mastectomy, *rNSM* robotic NSM, *BMI* body mass index, *DCIS* ductal carcinoma in situ, *IDC* invasive ductal carcinoma, *ILC* invasive lobular carcinoma, *SD* standard deviation, *NAC* nipple–areolar complex

The NSM procedure time per breast was longer for rNSM compared with open NSM (141.6 versus 83.9 min; *p* < 0.001), and the reconstruction procedure time per breast was also longer for rNSM (79.7 versus 62.5 min respectively; *p* = 0.008). The anesthesia time per patient, defined as “anesthesia start” until “anesthesia finish,” was longer for rNSM compared with open NSM (507.1 versus 294.8 min; *p* < 0.001) (Table 2). Outside of the roll-in phase, 14 surgeons performed more than one rNSM case and 7 surgeons performed more than three rNSM cases. Among surgeons performing at least three rNSMs, operative time decreased between the first and third case by 50.9 min (*p* = 0.03) from an average of 184.5 min to 133.6 min (Supplementary Table 1). There were no conversions from rNSM to open surgery. Estimated blood loss (EBL) was lower for rNSM (60.3 mL versus 97.8 mL, respectively; *p* = 0.03).

**Table 2:** Operative results

**Characteristic**	**rNSM**	**Open NSM**	***p*-Value**
Number of subjects	37	37	
Number of breasts	65	66	
NSM procedure time per breast, minutes*			
Mean (SD)	141.6 (76.2)	83.9 (48.5)	< 0.001
Range	18.0–495.0	15.0–222.0	
Reconstruction procedure time per breast, minutes*			
Mean (SD)	79.7 (39.9)	62.5 (31.4)	0.008
Range	27.0–278.0	16.0–157.0	
Anesthesia time per patient, minutes			
Mean (SD)	507.1 (136.2)	294.8 (89.7)	< 0.001
Range	304.0–888.0	145.0–476.0	
Conversion to open	0	NA	
EBL, mL			
Mean (SD)	60.3 (54.6)	97.8 (89.4)	0.033
Range	5.0–300.0	20.0–405.0	
PSM	6 (16.2)	6 (16.2)	> 0.9
Number of PSM due to “ink on tumor”	0 (0)	1 (2.7)	> 0.9
Number of PSM due to DCIS < 2 mm	6 (16.2)	5 (13.5)	> 0.9

^*^Analysis performed at breast level

Numbers are No. (%) unless indicated otherwise

*rNSM* robotic nipple-sparing mastectomy, *EBL* estimated blood loss, *NAC* nipple–areolar complex, *PSM* positive surgical margin, *DCIS* ductal carcinoma in situ

The trial-defined PSM rate was 16.2% for both groups. For rNSM patients, 0/37 patients had “ink on tumor” and 6/37 patients had DCIS ≤ 2 mm from the margin (PSM was located at the superficial margin in 5 patients, and DCIS was close to both the superficial and posterior margin in 1 patient). For open NSM patients, 1/37 had “ink on tumor” (PSM was located at the posterior margin), and 5/37 patients had DCIS ≤ 2 mm from the margin (PSM was located at the superficial margin in 2 patients, the posterior margin in 1 patient, and the nipple margin in 1 patient, and DCIS was close to both the superficial and posterior margin in 1 patient). No re-excisions were performed; however, the patient in the open NSM group with “ink on tumor” underwent postmastectomy radiation therapy.

Tissue expander placement was performed for reconstruction in 25/65 (38.5%) rNSM patients and 27/65 (41.5%) open NSM patients. Direct-to-implant reconstruction was performed in 40/65 (61.5%) rNSM patients and 38/65 (58.5%) open NSM patients. All implants were placed in the pre-pectoral plane and included structural support mesh, either biologic or synthetic. SPY was used to assess mastectomy flap viability at 7 of the 14 trial sites per the plastic surgeons’ discretion.

By the 42-day follow-up visit, unplanned reoperations occurred in two rNSM patients and eight open NSM patients (*p* = 0.085) and re-admissions occurred in zero rNSM patients and two open NSM patients (Table 3). There was no difference in NAC preservation at 42 days (100% for rNSM versus 98.5% for open NSM; *p* > 0.9). In both groups, there was no evidence of full-thickness necrosis of the NAC or mastectomy skin flap. Partial-thickness necrosis was observed at the NAC (1/65, 1.5%), not in the mastectomy flap (0/65, 0.0%), in the rNSM group and at the NAC (2/65, 3.0%) or mastectomy skin flap (3/65, 4.6%) in the open NSM group. The rNSM patient with partial-thickness necrosis was managed with wound care. One of the open NSM patients with NAC necrosis required surgical removal of the NAC reported at the 42-day visit, and the other was managed with wound care. One of the open NSM patients with skin flap necrosis required a reoperation reported at the 6-month visit, and the other two were managed with wound care. The number of patients who completed 6 months of follow-up was similar between groups (29/37 rNSM and 30/37 open NSM), and there were no cancer recurrences or deaths in either group at the 6-month follow-up visit.

**Table 3:** Outcomes at 42-day follow-up visit

**Characteristic**	**Overall**	**rNSM**	**95% CI**	**Open NSM**	**95% CI**
Number of subjects	74	37		37	
Reoperation	10/74 (13.5%)	2/37 (5.4%)	0.94%, 20%	8/37 (21.6%)	10%, 39%
Readmission	2/74 (2.7%)	0/37 (0.0%)	0.00%, 12%	2/37 (5.4%)	0.94%, 20%
Adjuvant treatment	26/74 (35.1%)	11/37 (29.7%)		15/37 (40.5%)	
Chemotherapy	5/26 (19.2%)	0/11 (0.0%)	0.00%, 32%	5/15 (33.3%)	13%, 61%
Radiation	1/26 (3.8%)	1/11 (9.1%)	0.48%, 43%	0/15 (0.0%)	0.00%, 25%
Hormonal	14/26 (53.8%)	7/11 (63.6%)	32%, 88%	7/15 (46.7%)	22%, 73%
Immunotherapy	2/26 (7.7%)	1/11 (9.1%)	0.48%, 43%	1/15 (6.7%)	0.35%, 34%
Other	4/26 (15.4%)	2/11 (18.2%)	3.2%, 52%	2/15 (13.3%)	2.3%, 42%
Completed follow-up, breasts	131/131 (100.0%)	65/65 (100.0%)		66/66 (100.0%)	
NAC preservation	130/131 (99.2%)	65/65 (100.0%)	93%, 100%	65/66 (98.5%)	91%, 100%
SKIN score for NAC, depth*					
A	127/130 (97.7%)	64/65 (98.5%)	91%, 100%	63/65 (96.9%)	88%, 99%
B	2/130 (1.5%)	1/65 (1.5%)	0.08%, 9.4%	1/65 (1.5%)	0.08%, 9.4%
C	1/130 (0.8%)	0/65 (0.0%)	0.00%, 7.0%	1/65 (1.5%)	0.08%, 9.4%
D	0/130(0.0%)	0/65(0.0%)	0.00%,7.0%	0/65(0.00%)	0.00%, 7.0%
Missing value	1	0		1	
SKIN score for breast skin, depth*					
A	127/130 (97.7%)	65/65 (100.0%)	93%, 100%	62/65 (95.4%)	86%, 99%
B	1/130 (0.8%)	0/65 (0.0%)	0.00%, 7.0%	1/65 (1.5%)	0.08%, 9.4%
C	2/130 (1.5%)	0/65 (0.0%)	0.00%, 7.0%	2/65 (3.1%)	0.53%, 12%
D	0/130(0.0%)	0/65(0.0%)	0.00%,7.0%	0/65(0.0%)	0.00%, 7.0%
Missing	1	0		1	
SKIN score for NAC, surface area**					
1	127/130 (97.7%)	64/65 (98.5%)	91%, 100%	63/65 (96.9%)	88%, 99%
2	1/130 (0.8%)	1/65 (1.5%)	0.08%, 9.4%	0/65 (0.0%)	0.00%, 7.0%
3	2/130 (1.5%)	0/65 (0.0%)	0.00%, 7.0%	2/65 (3.1%)	0.53%, 12%
4	0/130(0.0%)	0/65(0.0%)	0.00%,7.0%	0/65(0.0%)	0.00%,7.0%
Missing	1	0		1	
SKIN score for breast skin, surface area**					
1	127/130 (97.7%)	65/65 (100.0%)	93%, 100%	62/65 (95.4%)	86%, 99%
2	3/130 (2.3%)	0/65 (0.0%)	0.00%, 7.0%	3/65 (4.6%)	1.2%, 14%
3	0/130 (0.0%)	0/65 (0.0%)	0.00%,7.0%	0/65 (0.0%)	0.00%,7.0%
4	0/130(0.0%)	0/65(0.0%)	0.00%,7.0%	0/65(0.0%)	0.00%,7.0%
Missing	1	0		1	
Disease recurrence at 6 months	0/74 (0.0%)	0/29 (0.0%)	0.00%, 8.9%	0/30 (0.0%)	0.00%, 8.3%
Mortality	0/74 (0.0%)	0/37 (0.0%)	0.00%, 12%	0/37 (0.0%)	0.00%, 12%

Numbers are *n*/*N* (%) unless otherwise indicated

^*^Depth of mastectomy skin flap necrosis (MSFN) score definition

A = None, no evidence of MSFN

B = Color change of skin flap suggesting impaired perfusion or ischemic injury (may be cyanosis or erythema)

C = Partial-thickness skin flap necrosis resulting in at least epidermal sloughing

D = Full-thickness skin flap necrosis

^**^Surface area of MSFN score definition

1 = 0% area = none

2 = 1–10% area = breast, change affects 1–10% of breast skin

NAC, change affects 1–10% of nipple–areolar complex

3 = 11–30% area = breast, change affects 11–30% of breast skin NAC, 11–30% of NAC affected, or total nipple involvement

4 = > 30% area = change affects > 30% of breast skin or > 30% of NAC

*CI* confidence interval, *NAC* nipple–areolar complex, *SKIN* Skin Ischemia and Necrosis score

SAEs occurred in three rNSM patients (8.1%) and eight open NSM patients (21.6%) (*p* = 0.19) by the 42-day postoperative visit (Table 4). In the rNSM group, there were two hematomas and one pneumothorax. In the open NSM group, there was one hematoma, five infections associated with the implant or tissue expander, one seroma, and one wound dehiscence. None of the SAEs in the rNSM group were deemed to be device-related by the DSM.

**Table 4:** Serious adverse events at 42 days postoperative

Study Group	SAE	Timepoint	Intervention	Explanation
Open NSM	Infection of implant or TE	42 days	Reoperation	Return to OR for washout and implant exchange
Open NSM	Seroma	42 days	Reoperation	Return to OR for washout and implant exchange
Open NSM	Infection of implant or TE	42 days	Reoperation	Return to OR for washout and implant removal
rNSM	Hematoma	Post-op to discharge	Reoperation	Return to OR for washout and implant exchange
Open NSM	Hematoma	14 days	Reoperation	Return OR for control of bleeding and TE exchange
Open NSM	Infection of implant or TE	42 days	Reoperation	Return to OR for washout and implant removal
Open NSM	Wound dehiscence	42 days	Reoperation	Return to OR for revisions and eventual implant removal
Open NSM	Wound dehiscence	42 days	Reoperation	Return to OR for revisions and eventual implant removal
rNSM	Pneumothorax	Post-op to discharge	Prolonged hospitalization	Managed conservatively with inpatient monitoring
Open NSM	Infection of implant or TE	42 days	Reoperation	Return to OR for washout and TE removal
rNSM	Hematoma	Post-op to discharge	Reoperation	Return to OR for evacuation of hematoma
Open NSM	Infection of implant or TE	14 days	Reoperation	Return to OR for washout and implant removal

*SAE* serious adverse event, *rNSM* robotic nipple-sparing mastectomy, *TE* tissue expander

Changes in Breast-Q scores from baseline to 6 months postoperatively were more favorable, but not statistically different, in the rNSM group compared with the open NSM group in the scales of Sexual Well-Being (−7.2 versus −11.6, *p* = 0.2), Satisfaction with Breasts (−0.6 versus −6.3, *p* = 0.3), and Physical Well-Being of the chest (−9.1 versus −16.6, *p* = 0.10) (Table 5). NAC sensitivity using a 5-point scale was assessed at 42 days, 6 months and 1 year postoperatively. No statistically significant differences were observed across NAC-related outcomes, although differences favored rNSM in several domains (Table 6).

**Table 5:** BREAST-Q scores at baseline and 6 months postoperative

	Baseline			6-months		
	rNSM	Open NSM	p-value	rNSM	Open NSM	*p*-value
*Psychosocial well-being*						
Mean (SD)	84.2 (16.62)	76.5 (18.57)	0.062	76.6 (19.09)	74.2 (18.54)	0.8
Paired difference from baseline				-6.9 (19.48)	-1.9 (19.41)	0.6
*p*-value compared to baseline				0.12	0.6	
*Sexual well-being*						
Mean (SD)	70.9 (19.93)	70.6 (19.77)	0.7	62.6 (21.73)	59.2 (19.47)	0.4
Paired difference from baseline				-7.2 (21.59)	-11.6 (14.42)	0.2
p-value compared to baseline				0.12	0.045	
*Satisfaction with breasts*						
Mean (SD)	74.1 (19.80)	71.5 (21.33)	0.5	71.6 (19.78)	68.3 (15.55)	0.5
Paired difference from baseline				-0.6 (22.56)	-6.3 (23.40)	0.3
p-value compared to baseline				0.7	0.8	
*Physical well-being: chest*						
Mean (SD)	91.8 (10.67)	90.5 (12.86)	0.8	80.9 (18.17)	75.2 (14.99)	0.13
Paired difference from baseline				-9.1 (16.03)	-16.6 (16.98)	0.10
p-value compared to baseline				0.012	<0.001	
*Satisfaction with back*						
Mean (SD)	5.8 (2.07)	5.1 (0.59)	0.049	7.6 (4.19)	9.0 (4.56)	0.14
Paired difference from baseline				1.9 (4.24)	3.9 (4.55)	0.042
p-value compared to baseline				0.017	<0.001

**Table 6:** NAC scores at 42 days and 6 months postoperative

	**42 days**			**6 months**		
**Characteristic**	rNSM *N* = 33^1^	Open NSM *N* = 32^1^	*p*-Value^2^	rNSM *N* = 26^1^	Open NSM *N* = 25^1^	*p*-Value^2^
1. NAC sensitivity compared with before the operation			0.6			0.5
1- Insensitive	9/31 (29.0%)	12/32 (37.5%)		10/25 (40.0%)	13/24 (54.2%)	
2- Less sensitive	16/31 (51.6%)	14/32 (43.8%)		11/25 (44.0%)	8/24 (33.3%)	
3- The same	4/31 (12.9%)	2/32 (6.3%)		2/25 (8.0%)	3/24 (12.5%)	
4- Very sensitive	2/31 (6.5%)	4/32 (12.5%)		2/25 (8.0%)	0/24 (0.0%)	
*2. Significance of breast for sexuality*						
2a. Before operation			0.7			0.4
1- Unimportant	2/33 (6.1%)	1/32 (3.1%)		2/25 (8.0%)	2/25 (8.0%)	
2- Not very important	4/33 (12.1%)	3/32 (9.4%)		4/25 (16.0%)	0/25 (0.0%)	
3- Neither important nor unimportant	5/33 (15.2%)	6/32 (18.8%)		4/25 (16.0%)	5/25 (20.0%)	
4- Important	16/33 (48.5%)	12/32 (37.5%)		12/25 (48.0%)	14/25 (56.0%)	
5- Very important	6/33 (18.2%)	10/32 (31.3%)		3/25 (12.0%)	4/25 (16.0%)	
2b. Now			> 0.9			0.7
1- Unimportant	2/32 (6.3%)	4/32 (12.5%)		2/25 (8.0%)	4/25 (16.0%)	
2- Not very important	4/32 (12.5%)	5/32 (15.6%)		4/25 (16.0%)	2/25 (8.0%)	
3- Neither important nor unimportant	9/32 (28.1%)	9/32 (28.1%)		4/25 (16.0%)	7/25 (28.0%)	
4- Important	13/32 (40.6%)	10/32 (31.3%)		14/25 (56.0%)	11/25 (44.0%)	
5- Very important	4/32 (12.5%)	4/32 (12.5%)		1/25 (4.0%)	1/25 (4.0%)	
*3. Significance of NAC for sexuality*						
3a. Before operation			0.6			0.2
1- Unimportant	2/32 (6.3%)	1/32 (3.1%)		4/25 (16.0%)	2/25 (8.0%)	
2- Not very important	3/32 (9.4%)	3/32 (9.4%)		3/25 (12.0%)	0/25 (0.0%)	
3- Neither important nor unimportant	8/32 (25.0%)	11/32 (34.4%)		4/25 (16.0%)	10/25 (40.0%)	
4- Important	15/32 (46.9%)	10/32 (31.3%)		10/25 (40.0%)	9/25 (36.0%)	
5- Very important	4/32 (12.5%)	7/32 (21.9%)		4/25 (16.0%)	4/25 (16.0%)	
3b. Now			0.2			0.6
1- Unimportant	2/32 (6.3%)	4/32 (12.5%)		4/25 (16.0%)	5/25 (20.0%)	
2- Not very important	3/32 (9.4%)	6/32 (18.8%)		5/25 (20.0%)	2/25 (8.0%)	
3- Neither important nor unimportant	12/32 (37.5%)	11/32 (34.4%)		9/25 (36.0%)	9/25 (36.0%)	
4- Important	13/32 (40.6%)	6/32 (18.8%)		6/25 (24.0%)	9/25 (36.0%)	
5- Very important	2/32 (6.3%)	5/32 (15.6%)		1/25 (4.0%)	0/25 (0.0%)	
4. Did sexual pleasure change since the operation because of loss of NAC sensitivity?			0.10			0.3
1- Absent	2/30 (6.7%)	5/30 (16.7%)		4/26 (15.4%)	2/25 (8.0%)	
2- Decreased a lot	5/30 (16.7%)	3/30 (10.0%)		3/26 (11.5%)	4/25 (16.0%)	
3- Substantial decrease	1/30 (3.3%)	7/30 (23.3%)		3/26 (11.5%)	2/25 (8.0%)	
4- A little decrease	13/30 (43.3%)	8/30 (26.7%)		6/26 (23.1%)	12/25 (48.0%)	
5- Unchanged	9/30 (30.0%)	7/30 (23.3%)		10/26 (38.5%)	5/25 (20.0%)	
5. Touching of the NAC is			> 0.9			0.7
1- Very unpleasant	1/33 (3.0%)	1/32 (3.1%)		1/26 (3.8%)	1/25 (4.0%)	
2- Unpleasant	3/33 (9.1%)	4/32 (12.5%)		3/26 (11.5%)	2/25 (8.0%)	
3- Neither pleasant nor unpleasant	26/33 (78.8%)	24/32 (75.0%)		22/26 (84.6%)	20/25 (80.0%)	
4- Pleasant	3/33 (9.1%)	3/32 (9.4%)		0/26 (0.0%)	2/25 (8.0%)	
6. Change of nipple reaction to cold or touch			0.4			0.3
1- No reaction	12/32 (37.5%)	15/32 (46.9%)		6/26 (23.1%)	8/25 (32.0%)	
2- A lot	5/32 (15.6%)	3/32 (9.4%)		4/26 (15.4%)	2/25 (8.0%)	
3- Weaker	6/32 (18.8%)	10/32 (31.3%)		11/26 (42.3%)	6/25 (24.0%)	
4- Hardly	6/32 (18.8%)	2/32 (6.3%)		4/26 (15.4%)	4/25 (16.0%)	
5- Unchanged	3/32 (9.4%)	2/32 (6.3%)		1/26 (3.8%)	5/25 (20.0%)	
7a. Satisfaction with position of NAC on the breast			0.7			0.7
1- Very unsatisfied	2/32 (6.3%)	4/31 (12.9%)		4/26 (15.4%)	3/25 (12.0%)	
2- Unsatisfied	3/32 (9.4%)	4/31 (12.9%)		4/26 (15.4%)	2/25 (8.0%)	
3- Neither satisfied nor unsatisfied	4/32 (12.5%)	6/31 (19.4%)		2/26 (7.7%)	4/25 (16.0%)	
4- Satisfied	15/32 (46.9%)	10/31 (32.3%)		8/26 (30.8%)	11/25 (44.0%)	
5- Very satisfied	8/32 (25.0%)	7/31 (22.6%)		8/26 (30.8%)	5/25 (20.0%)	
7b. Not satisfied (score 1–3 in a) because NAC is too.			0.10			0.3
1- Lateral	6/31 (19.4%)	1/31 (3.2%)		3/25 (12.0%)	0/24 (0.0%)	
2- Caudal	1/31 (3.2%)	3/31 (9.7%)		2/25 (8.0%)	2/24 (8.3%)	
3- Cranial	0/31 (0.0%)	1/31 (3.2%)				
4- Medial	1/31 (3.2%)	0/31 (0.0%)				
5- N/A	23/31 (74.2%)	26/31 (83.9%)		20/25 (80.0%)	22/24 (91.7%)	
8. Would choose the same operation again			0.7			0.3
1- Certainly not	1/33 (3.0%)	1/32 (3.1%)		3/26 (11.5%)	0/25 (0.0%)	
2- Probably not	1/33 (3.0%)	0/32 (0.0%)		1/26 (3.8%)	1/25 (4.0%)	
3- Maybe	2/33 (6.1%)	4/32 (12.5%)		1/26 (3.8%)	3/25 (12.0%)	
4- Probably	7/33 (21.2%)	4/32 (12.5%)		3/26 (11.5%)	6/25 (24.0%)	
5- Certainly	22/33 (66.7%)	23/32 (71.9%)		18/26 (69.2%)	15/25 (60.0%)	
9. Would advise this operation to other women			0.9			0.093
1- Certainly not	1/33 (3.0%)	0/32 (0.0%)		2/26 (7.7%)	0/25 (0.0%)	
2- Probably not	0/33 (0.0%)	0/32 (0.0%)		2/26 (7.7%)	0/25 (0.0%)	
3- Maybe	4/33 (12.1%)	4/32 (12.5%)		0/26 (0.0%)	2/25 (8.0%)	
4- Probably	4/33 (12.1%)	6/32 (18.8%)		4/26 (15.4%)	8/25 (32.0%)	
5- Certainly	24/33 (72.7%)	22/32 (68.8%)		18/26 (69.2%)	15/25 (60.0%)	

^1^Numbers are shown as *n*/*N* (%), where *n* represents the number of patients selecting a given response and *N* represents the number of patients who provided a response to that domain

^2^Fisher’s exact test

*rNSM* robotic nipple-sparing mastectomy

## Discussion

In this randomized trial of patients with early stage breast cancer, rNSM was technically feasible, and the safety profile was comparable to open NSM. NAC preservation was excellent in both groups, and EBL was significantly lower with rNSM. Rates of unplanned reoperation and SAEs were lower in the rNSM group, but the differences were not statistically significant. No SAEs in the rNSM cohort were attributed to the study device. The SAE rate observed in this study is at the lower end of reported NSM with implant-based reconstruction SAE data and is likely due to experienced breast surgeons and careful patient selection.^14^ Importantly, oncologic outcomes were similar, with no difference in PSM, no cases of ink on tumor in the rNSM group, and no cancer recurrences observed in either group at early follow-up.

The operative times for both the mastectomy procedure and reconstruction were longer for rNSM patients compared with open NSM, consistent with the finding from a prior randomized trial evaluating rNSM.^15^ Some of these differences likely reflect the learning curve associated with adoption of a new surgical platform. However, there is also time associated with positioning and docking the robot and instrument insertions. Robotic breast reconstruction may increase in utilization in the future, with increased adoption of rNSM and operative times likely being a point of interest.^16^ Although the majority of surgeons in this study were newer to robotic surgery, they were experienced breast surgeons with open NSM. The operative times for rNSM decreased significantly between the first and third cases, highlighting significant improvement even in the very early stages of the learning curve. Loh and colleagues demonstrated a significant reduction in operative time for rNSM after 20–30 cases, supporting the need for structured, ongoing simulation training as surgeons incorporate rNSM into clinical practice.^17^

Patient-reported outcomes are essential to the evaluation of breast surgery and reconstruction. At the 6-month follow-up, changes in BREAST-Q scores were more favorable in the rNSM group compared with open NSM across multiple domains, including Sexual Well-Being, Satisfaction with Breasts, and Physical Well-Being of the chest, although these differences were not statistically significant. Our findings do not demonstrate statistically significant differences in sensation; however, observed numerical patterns and prior literature suggest this may warrant further evaluation.^18^ Improved sensation may be due to the reduced force with retraction, using CO_2_ insufflation compared with conventional metal or plastic retractors used in open NSM. In addition, enhanced visualization and precise instrumentation associated with the robotic approach may allow for improved preservation of sensory nerves supplying the NAC.^19^ Patients undergoing rNSM more frequently reported that they would choose the same operation again and recommend it to other women, although this difference was not statistically significant.

Despite these strengths, this study has important limitations to consider when interpreting the results. The average age of our patients (46–49 years old) is younger than the average age of patients diagnosed in the USA (62 years old).^20^ Secondary to our relatively conservative eligibility criteria, the study populations had lower average BMI, moderate breast size and ptosis, and small tumors. Patients with previous breast implants, diabetes, and/or active smokers were not eligible for the trial. Patients outside of the study criteria could be expected to have higher complication rates and longer operative times. Thoughtful consideration should be used when expanding rNSM indications.

Patients were not blinded to their procedure, and this may have introduced bias in the patient-reported outcomes. Our results are underpowered because trial enrollment was prematurely terminated owing to FDA 510(k) clearance of the da Vinci SP^®^ Robotic platform for NSM. Long-term oncologic outcomes for our patients are not yet available and will be reported in future publications.

In conclusion, rNSM using the da Vinci SP^®^ system was feasible and demonstrated a safety profile comparable to open NSM, with similar margin status and early postoperative outcomes. While serious adverse events and patient-reported outcomes were more favorable in the rNSM group, these differences were not statistically significant. Operative times were longer with rNSM, likely reflecting early experience with the technique, and are expected to improve over time. The adoption of any new surgical technology raises important questions regarding the feasibility of its broader dissemination. Factors such as costs, operating room availability, and access to the SP robotic platform may limit the widespread implementation of rNSM. These considerations warrant careful and critical evaluation.

These findings support the safe adoption of rNSM in carefully selected patients, following structured training in experienced centers. Ongoing follow-up to better define long-term oncologic outcomes will continue.

## Supplementary Information

Below is the link to the electronic supplementary material.

Supplementary file1 (DOCX 233 KB)

## Data Availability

Individual de‑identified participant data and the associated data dictionary are held by the study sponsor. These data are not publicly available and may be accessed only with written permission from the sponsor.
