# Supplementary material for: A Prospective, Multicenter Randomized Controlled Trial (RCT) of the da Vinci SP® Surgical System versus Open Surgery in Nipple-Sparing Mastectomy (NSM) Procedures for Patients with Breast Cancer
Source: Ann Surg Oncol. 2026 Jul 16;33(11):9662–73. doi: 10.1245/s10434-026-20201-6 (PMC13630962; doi:10.1245/s10434-026-20201-6)
Supplement: Supplementary file 1 [file 10434_2026_20201_MOESM1_ESM.docx]

# Supplementary Figure 1. Study Eligibility Criteria

Inclusion and exclusion criteria for patient eligibility. Criteria are listed verbatim from the study protocol.

## Inclusion Criteria

1. Female sex
2. BMI < 30
3. Age greater than 21
4. Candidate for a unilateral or bilateral nipple-sparing mastectomy (NSM) procedure with immediate one- or two-stage prosthetic implant-based reconstruction
5. Existing diagnosis of DCIS and/or invasive breast carcinoma clinical stage Tis, T1, T2 with N0
6. Tumor-skin distance of ≥ 5 mm (confirmed by all imaging modalities performed which may include but are not limited to mammography, ultrasound, MRI assessments or physical exam)
7. Tumor(s) located ≥ 1 cm from the NAC (confirmed by all imaging modalities performed which may include but are not limited to mammography, ultrasound, MRI assessments or physical exam)
8. Calcifications of ≤ 5 cm for DCIS
9. Breast ptosis ≤ Grade 2
10. Breast cup size C or smaller
11. Low to moderate risk for anesthesia (ASA class I, II or III)
12. Willing and able to provide written informed consent
13. Willing and able to comply with the study protocol requirements including study follow-up visits according to the schedule of the study up to the final visit at 5 years postoperatively

## Exclusion Criteria

14. Prior history of contralateral breast malignancy (invasive disease or DCIS) less than 5 years prior to the study entry
15. Previous extensive breast surgery of the ipsilateral breast (Note: needle or core biopsies, and excisional biopsies performed at least one year prior to study are allowed)
16. Inflammatory and/or infectious skin condition and/or unhealed wounds on the ipsilateral breast
17. Known diagnosis of metastatic or inflammatory breast cancer
18. Neoadjuvant chemotherapy for breast cancer within 4 weeks prior to study procedure
19. Prior radiation treatment to the ipsilateral breast or chest
20. Plan (at time of screening) for other concomitant procedures during the same hospital admission (e.g., oophorectomy, hysterectomy, etc.) (Note: concomitant contralateral prophylactic NSM procedures, port placement or removal, axillary lymph node surgery, and neurotization procedures are allowed)
21. Current diagnosis of other primary cancer(s) or diagnosis of synchronous primary tumor (Note: non-melanoma skin cancers that are not located on the breast are allowed)
22. Current (at time of screening) clinical diagnosis of or suspected Paget’s disease of the nipple
23. Current tobacco usage (e.g., smoking, vaping, etc.) within 6 weeks prior to the study procedure
24. Known history of diabetes with hemoglobin A1c levels ≥ 8
25. Current high risk for anesthesia (ASA class ≥ IV) or significant existing medical comorbidities (i.e., cardiac, pulmonary and neurologic) that preclude longer anesthesia times
26. Other contraindication for general anesthesia or surgery
27. Known bleeding or clotting disorder
28. Pregnant or suspected to be pregnant, or currently breastfeeding

**Supplementary Table 2. Operative times for rNSM procedure for first and third case**.

| **Characteristic** | **1^st^ Case** | **3^rd^ Case** | **p-value^1^** |
| --- | --- | --- | --- |
| Number of rNSM procedures | 22 | 12 | 0.03 |
| Operative time, mean; minutes (SD)^2^ | 184.5 (56.9) | 133.6 (52.8) |  |
| Operative time, range; minutes | 110.0 - 287.0 | 58.0 - 232.0 |  |
| ^1^Wilcoxon rank sum test  ^2^Data are pooled from all surgeons who completed at least 3 cases and includes SP roll-in cases if applicable. If a subject had bilateral mastectomy, the average time for both breasts was recorded.  rNSM, robotic nipple-sparing mastectomy | | | |
